# Financial Risk Management and Explainable, Trustworthy, Responsible AI

**DOI:** 10.3389/frai.2022.779799

**Published:** 2022-02-28

**Authors:** Sebastian Fritz-Morgenthal, Bernhard Hein, Jochen Papenbrock

**Affiliations:** ^1^Bain & Company, Frankfurt, Germany; ^2^Ernst & Young, Munich, Germany; ^3^NVIDIA GmbH, Würselen, Germany

**Keywords:** risk management, EU AI act, artificial intelligence, machine learning, financial regulation and compliance, explainable AI

## Abstract

This perspective paper is based on several sessions by the members of the Round Table AI at FIRM^1^, with input from a number of external and international speakers. Its particular focus lies on the management of the model risk of productive models in banks and other financial institutions. The models in view range from simple rules-based approaches to Artificial Intelligence (AI) or Machine learning (ML) models with a high level of sophistication. The typical applications of those models are related to predictions and decision making around the value chain of credit risk (including accounting side under IFRS9 or related national GAAP approaches), insurance risk or other financial risk types. We expect more models of higher complexity in the space of anti-money laundering, fraud detection and transaction monitoring as well as a rise of AI/ML models as alternatives to current methods in solving some of the more intricate stochastic differential equations needed for the pricing and/or valuation of derivatives. The same type of model is also successful in areas unrelated to risk management, such as sales optimization, customer lifetime value considerations, robo-advisory, and other fields of applications. The paper refers to recent related publications from central banks, financial supervisors and regulators as well as other relevant sources and working groups. It aims to give practical advice for establishing a risk-based governance and testing framework for the mentioned model types and discusses the use of recent technologies, approaches, and platforms to support the establishment of responsible, trustworthy, explainable, auditable, and manageable AI/ML in production. In view of the recent EU publication on AI, also referred to as the EU Artificial Intelligence Act (AIA), we also see a certain added value for this paper as an instigator of further thinking outside of the financial services sector, in particular where “High Risk” models according to the mentioned EU consultation are concerned.

## Introduction

The European Commission is proposing one of the first laws globally to regulate the use of artificial intelligence. AIA is a cross-sectoral regulation of AI, which addresses particularly governance requirements around so-called high-risk AI systems, and which more generally recommends the adoption of principles in the spirit of creating trustworthy AI. Interestingly, credit scoring models are explicitly given as an example of a high-risk use case.

Pursuant to the requirements of the AIA and existing supervisory expectations, those pursuing an AI-first bank/strategy must be equipped with suitable risk management as well as suitable infrastructure and technology (RiskTech, TrustTech, Algo Audit, Regulatory Sandbox).

In this context, the considerations of fairness and explainability should in principle be applied to all types of models, not just AI, but they are amplified for AI because of their higher complexity and a certain level of in-transparency of more complex algorithms. In this respect, we propose that the requirements for explainability and fairness should, as a leading principle, depend on the application purpose of a model rather than on the choice of its model design. A model for automating credit decisions, for example, the results of which affect human lives and are publicly visible, should ideally be free of unwanted bias and meet requirements for model transparency even if it is a classical model. In some legislations, credit customers even have the explicit right to request an explanation of the reasons behind credit decisions pertaining to themselves, even though the depth of the explanation may depend on whether the actual decision was to the positive or to the negative.

This article focuses on the financial sector because this sector has been in a pioneering position regarding regulation that covers the mentioned topics of explainability and fairness. However, the recent EU proposal for an AIA identifies further use cases and industry sectors in which a certain need for compliance with similar requirements is to be expected for the future. In this respect, we believe that the experience from the financial services sector should be profoundly valuable also for other industries sooner rather than later.

This paper is structured as follows:

In section Risk Management and AI, we provide a high-level description of use cases, based on actually existing AI technology within risk management functions of financial institutions and describe how we expect financial services regulators to approach these models. This view is the result of our discussion at the FIRM Working Group and also takes into account a Policy Discussion Paper of Deutsche Bundesbank (Deutsche Bundesbank, 2020).

In section Fairness and Bias, we describe the challenge of potential model bias, and why it is important to work toward fair decision-making algorithms, as stated by the Ethics Guidelines for Trustworthy AI (Independent High-Level Expert Group on Artificial Intelligence, 2019). We start with a definition of bias, explain the principle of fairness, and provide a procedure to test against or validate the potential (un-)fairness of a decision-making algorithm.

In our view, fairness is a pre-condition to develop and run algorithms the decisions of which can be trusted. As a consequence, in section Addressing Trustworthy AI With Technology, we describe how we believe the Trustworthiness of AI models can be assessed. This includes privacy-preserving methods as well as technologies for explainability and transparency. Further, we offer an approach as to how Trustworthy AI can be optimized within a set of multiple objectives. We conclude the section with references to further reading.

In section A European Use Case for Explainable AI in Credit Risk Management, we discuss a specific model in the credit risk management of a major European insurance group, as a tangible use case of explainable AI. Transparency and explainability were prioritized during the build of this model, and both were achieved using SHAP (Shapley Additive exPlanations, as described in section Addressing Trustworthy AI With Technology and references therein).

We conclude with seven key takeaways in section Conclusion.

## Risk Management and AI

Our focus in this article is on Financial Services (i.e., regulated entities, but not necessarily fully regulated areas of application), models used in (or close to) regulatory pillar 1 and pillar 2 risk measurement and prediction for risk or economic capital allocation, accounting, compliance (Anti Money Laundering, Fraud Detection, Transaction Monitoring), client classification/credit decisioning, collections optimization, early warning systems, etc. Of course, the generic outcomes of this paper will just as well apply to models that are more remote from risk management, such as robo advisors, customer lifetime value related recommender models, client coverage etc., to the extent that a financial services entity sees value in them.

We assume that models would need to be ready to undergo regular supervisory or statutory or internal auditor review, and in certain instances of pillar 1 modeling even need to receive supervisory approval. In the absence of more complete and detailed guidance for model risk and model governance of other model types, the requirements for pillar 1 approved internal models have frequently received the largest amount of attention when it came to developing model governance frameworks in the past. Benchmarking results across the market indicate that this realm is therefore the one with the most mature status quo, shaped by long standing and continuously developing supervisory scrutiny, and at the same time by the need to streamline to a business practice that seems viable under profitability considerations.

Our starting point is therefore to apply a typical regulatory model approval process as a blueprint. We focus on model approval because from the perspective of what we are interested in, both development, initial and (guidelines for) periodic validation, and the associated model change processes are assessed during model approval. We also want to address which additional requirements (over and above standard models) AI/ML models would most likely need to fulfill for regulatory approval.

Given the considerations around the model change processes of regulated pillar 1 models we will explore static models first. After that, the generalization to self-learning/self-modifying models could happen theoretically through an iterative application of the above-mentioned model approval process, under certain additional efficiency requirements. Given efficiency constraints, the entailing effort will effectively limit the frequency in which such model changes can be afforded, unless the materiality of the model changes can be monitored in some way and a layered process can be introduced that limits effort for immaterial changes.

The classification and measurement of this materiality of model changes is also at the core of the regulatory/supervisory model change process, and might allow for a first step toward supervisory acceptance of self-learning models in case, e.g., the self-learning process can be steered to an extent where only immaterial model changes (according to supervisory definition) are suggested, and then carried through under appropriate governance. The authors doubt though, that a self-learning process that effectively produces material model changes would be accepted in the more strictly regulated areas of application in the foreseeable future, particularly in pillar 1 capital models.

Depending on their application however, the authors hold the view that self-learning models should not generally be discouraged. Particularly:

- Within anti financial crime, anti-money laundering and fraud detection methods, there is tangible competition between fraudsters on the one hand and methods to detect them and prevent their deeds on the other. A targeted introduction of self-learning models in this space could therefore lead to better prevention. Compared to currently (still) wide-spread methods, especially in anti-money laundering (i.e., decision trees and expert based systems), these models have proven their capability to significantly decrease false alarm rates. This might outweigh the need for model stability from other areas of application of models.
- Also, for trading models (including algo trading/robo advice), as well as models to decide about capital and liquidity allocation optimization, the authors believe that self-learning capabilities might be desirable from an overall perspective. However, given that these models are used for economic decision making, portfolio allocation etc., the depth of their explainability to different stakeholder groups will be of greater importance than for the first group of models above.
- More questionable, however, is the use of self-learning models for the calculation of regulatory capital for credit risk, credit decision making, client segmentation and classification, given the much higher transparency requirements for regulation and customer protection laws, as well as the different character and somewhat slower clock speed of classical credit risk.
- Generally, self-learning models are prone toward bias/drift over time. Hence, adequate validation methods and processes need to be applied to manage these issues.

## Fairness and Bias

Through a short discussion of the notions of bias and fairness and a high-level look at their interplay, the following section effectively makes a case for the need to keep (or make) models explainable. Besides potential bias and (un-)fairness, the level of differentiability and continuity as well as potential cliff effects and behavior under extreme parameter settings (stability concerns) of models also need to be well-understood. This adds further weight to a case for model explainability, even though neither of the mentioned aspects will be explored in more detail in this perspective paper.

### Definitions of Bias and Fairness

The Independent High-Level Expert Group on Artificial Intelligence (2019) states that bias is an inclination of prejudice toward or against a person, object, or position. Bias drives the value of many predictive models, such as most risk prediction models (wanted bias) but it can also be detrimental to it. Bias can be good or bad, intentional or unintentional. In certain cases, bias can result in unwanted discriminatory and/or unfair outcomes, labeled in this document as unfair bias.

Different from the notion of Bias, in all the following discussions, the notions of unfairness and fairness always refer to any chosen, mathematical definition of fairness an institution is applying with respect to a given model, based on ethical and strategic deliberations about the purpose to which the model in view is applied in this institution. Popular notions of fairness include demographic parity (also called statistical parity, e.g., blondes and brunettes have the same chance to get a loan), equalized odds (blondes and brunettes who all meet certain other requirements have the same chance to get a loan) or the well-calibratedness (among those who got a loan, blondes, and brunettes are equally represented as in any random sample). A more in-depth description can for example be found in Loftus et al. (2018), Zhang and Bareinboim (2018), and Hutchinson and Mitchell (2019).

Bias can arise in many ways in any statistical model. However, it may require more targeted efforts to be detected in AI systems. We see that at least the following distinct root causes for bias typically appear:

- Bias inherent in training data (e.g., certain populations underrepresented or certain populations with different coincidence structures to the model's target variable, data being “polluted” with erroneous entries, outliers, noise, or training data are sourced from past human decision-making which contained bias).
- Algorithmic bias (caused by characteristics of the methodology).

For example, in any data-driven statistical model, particularly an AI system such as one produced through machine learning, bias in data collection and training can result in this model or AI system demonstrating bias. In logic-based AI, such as a rule-based system, bias can be caused by the way in which a knowledge engineer might have viewed the rules that apply in a particular setting, and subsequently codified this view in a model. Any specific bias can also arise in a formerly bias-free model due to self-learning and adaptation through interaction. It can arise through personalization, whereby users are presented with recommendations or information feeds that are tailored to the tastes of other users form a seemingly comparable group, because this bias was present in the tastes of that comparison group. It does not necessarily relate to human bias or human-driven data collection. It can evolve, for example, through the limited contexts in which a system is used, in which case there is no opportunity to generalize it to other contexts. For a United States Fair Lending Perspective on Machine Learning (see Hall et al., 2021).

### The Principle of Fairness

During their development, deployment and use, AI systems which impact human lives should be fair in the sense that their output obeys a chosen, reasonable definition of fairness. Next to ethical deliberations, this will help to minimize reputational risk from reactions of or on behalf of adversely affected customers, or at least help to defend respective decisions. We acknowledge that there are different mathematical interpretations of fairness, which partly contradict each other and must be defined based on corporate strategy and ethics^2^.

This means that in principle, for each model with fairness requirements, a specific definition of fairness needs to be chosen with respect to each explanatory variable or model feature. However, we also observe that any concept of fairness entails both a substantive and a procedural dimension upon implementation. For the present paper, we will retreat to discussing these overarching aspects that are invariant under changes of the fairness definition. For this reason, it is enough to speak about “fairness” as though it was a clearly defined notion for the rest of this paper. The substantive dimension of fairness (i.e., independently of any chosen particular definition of fairness) implies a commitment to:

1. ensuring a fair (in some sense “equal and just”) distribution of both benefits and costs,
2. ensuring that the effect a model has on individuals and groups is free from unfair bias, discrimination, and stigmatization.

The procedural dimension of fairness entails the ability to contest and seek effective redress against decisions made by AI systems and by the human beings who operate them. To do so, the decision in question must have an accountable owner, this owner must be identifiable (effectively a human or a committee of humans in the relevant governance roles for the model in question), and the decision-making processes needs to be explicable.

Fairness and bias interplay in an important way where bias in the model becomes ethically disputable for at least one group of stakeholders to a given model. This could be the case for any one of the above-mentioned root causes of bias. A typical example would be the question of whether different health insurance premiums for men and women can be or should be derived from potentially measurable differences in actual cost causation in historical data. Similarly, suppose the training data for a credit pricing model say that kebab stands have historically failed significantly more often or less often than Currywurst stands; is a model that therefore provides correspondingly different interest rate conditions for the two discriminatory? A different societal fact of today is still that men earn more on average than women. It is an open question how this fact could proliferate into loan approval rates or into the risk premium on corresponding interest rates. However, any bank should be aware that this type of gender bias can easily arise in models even though gender itself is not an explanatory variable in the model. Here, each individual bank should clarify which bias is acceptable to it, and which definition of fairness it thinks it can justifiably pursue; it may be that a higher loan rejection rate for women would be statistically justifiable from the training data (and might comply with an equalized odds fairness definition), but a bank still would not want a corresponding model for ethical (or reputational) reasons.

### Technical Validation—Considerations

In our view, a typical full scope model validation including the requirement to test for fairness/unwanted bias fully covers what needs to be known about the models we have in mind. It should be able to uncover all more than immaterial model risk, independently of the question which model type is used. Keep in mind that performing the necessary tests, e.g., around conceptual soundness or around model plausibility, would of course entail a different technical need for validation analyses to be performed, depending on whether the model under review is more or less transparent or complex. For technical validation, we advocate that the following criteria be considered:

- An important first step is the preparation and cleansing of the respective training and test data sets. The focus of this is to identify potentially erroneous data entries, outliers, and noise; and to transform where possible, otherwise eliminate.
- The basic determination of the “model tests” should be derived solely from the problem to be solved and the use of the model results, and not from the properties of the model. Otherwise, there is a danger of overlooking undiscovered weaknesses of a particular methodology. For example, a credit decisioning model, discriminatory power, stability, calibration, explainability to non-expert stakeholders (e.g., credit applicants) and freedom of unwanted bias are relevant tests, and this does not depend on whether the model is a score card or a neural network. For an anti-money laundering alert model, calibration is far less important, while the false positive ratio frequently replaces a more general look at discriminatory power, etc.
- Areas that are more difficult to be tested (for example, historically unprecedented capital market scenarios) should be identified and considered when applying the model. In credit risk models, this corresponds to a (well-governed) override/overruling process, in accounting models this could be management overlays/top-level adjustments as we have frequently seen them through the current COVID related economic anomalies.
- The intensity with which a specific model is validated further depends on the specific model risk appetite. The amount of residual risk which the model user wants to accept should hence be explicitly stated and aligned with the general risk appetite of an institution.
- For models that support critical decisions (significant impact on individual customers, large monetary impact, etc.) and hence have a higher impact on reputational risk, a plausibility check or “explanation” of the results should be performed irrespective of whether they are inherently transparent or not. This explanation should identify the main drivers of the model results and make them understandable. While this basic task is again in our view independent of model type, the technicalities of its conduction are not. For example, in a linear model of scorecard type, the explanation is considerably easier to achieve than in an in-transparent and (likely) highly non-linear model. Achieving this explanation also for the mentioned non-linear and intransparent models is the goal of what we call “explainable AI.” Together with other aspects, Explainability, on the other hand, is an integral part of what we call “trustworthy AI”). Intensity again corresponds to risk appetite. This overall approach corresponds to the usual process of model risk management:
  1. Design a risk strategy and risk appetite
  2. Derive a model risk strategy and model risk appetite
  3. Perform a model risk assessment and categorization of models (e.g., low/medium/high risk)
  4. Declare appropriate risk mitigation measures (surcharges, buffers, increase of validation intensity, strengthening of internal controls and governance) or acceptance of risk.
  5. Ensure you can comply with legal requirements, e.g., rejected borrowers may have the right to be informed about reasons for rejection, depending on legislation.
- The auditability of the model and its decisions needs to be guaranteed to enable accountability for the model's results. Again, while this basic requirement holds for all model types, it will lead to more technical work for (e.g.,) self-learning models. Specifically, the full data history, parameters and meta-data of self-learning models must be archived either periodically at a frequency that is informed by the model's update frequency, or after each meaningful change of the model. In short: If I don't know what the (self-learning) model looked like yesterday, how can I reproduce yesterday's results?

The last-mentioned point about self-learning models is of course not the only aspect in which the validation of self-learning models delivers higher technical hurdles to answer the same basic questions as for other models. For example, if model stability is an aspect of model plausibility, then jumps in model parameters should be examined across model changes. If the model keeps updating itself, this will cause higher effort and will have to be done in a more automated way etc.

Generally speaking, a set of basic standard testing and validation procedures should be applied to all models.

However, one must differentiate between various use cases. Additionally, the model specific shape and effort to conduct the corresponding tests will vary depending on model type (e.g., if you know that it is reinforcement learning, then you know the mechanics and weaknesses of the approach). The more complex a model is, the more one must study its behavior and cannot do normal tests anymore. For example, it is quite common to use AI-based synthetic datasets to test the model.

To switch perspectives for once, the following list of technical efforts and base rules will likely have to be used to achieve the aims and targets of the general, basic tests in the case that more complex AI/ML models are used:

1. Create enhanced incident management: what to do if the AI model is broken
2. Create more capable infrastructure to observe and monitor potential model and/or data drift
3. Be more rigid around stressing the model (with human test data and AI-based synthetic data)
4. Design and implement the infrastructure for the complete historization of models, data, runtime environments
5. Design and carry through multi-criteria choice for the best model according to the Pareto optimum (efficiency line of the best trade-off relations between several, quite competing goals such as accuracy, transparency, fairness, explainability, energy consumption, while being compliant with data privacy and other regulations).
6. Conduct the identification of risks and side effects of integration into existing processes, requirements, and complexities for use, etc.

**Figure 1:**
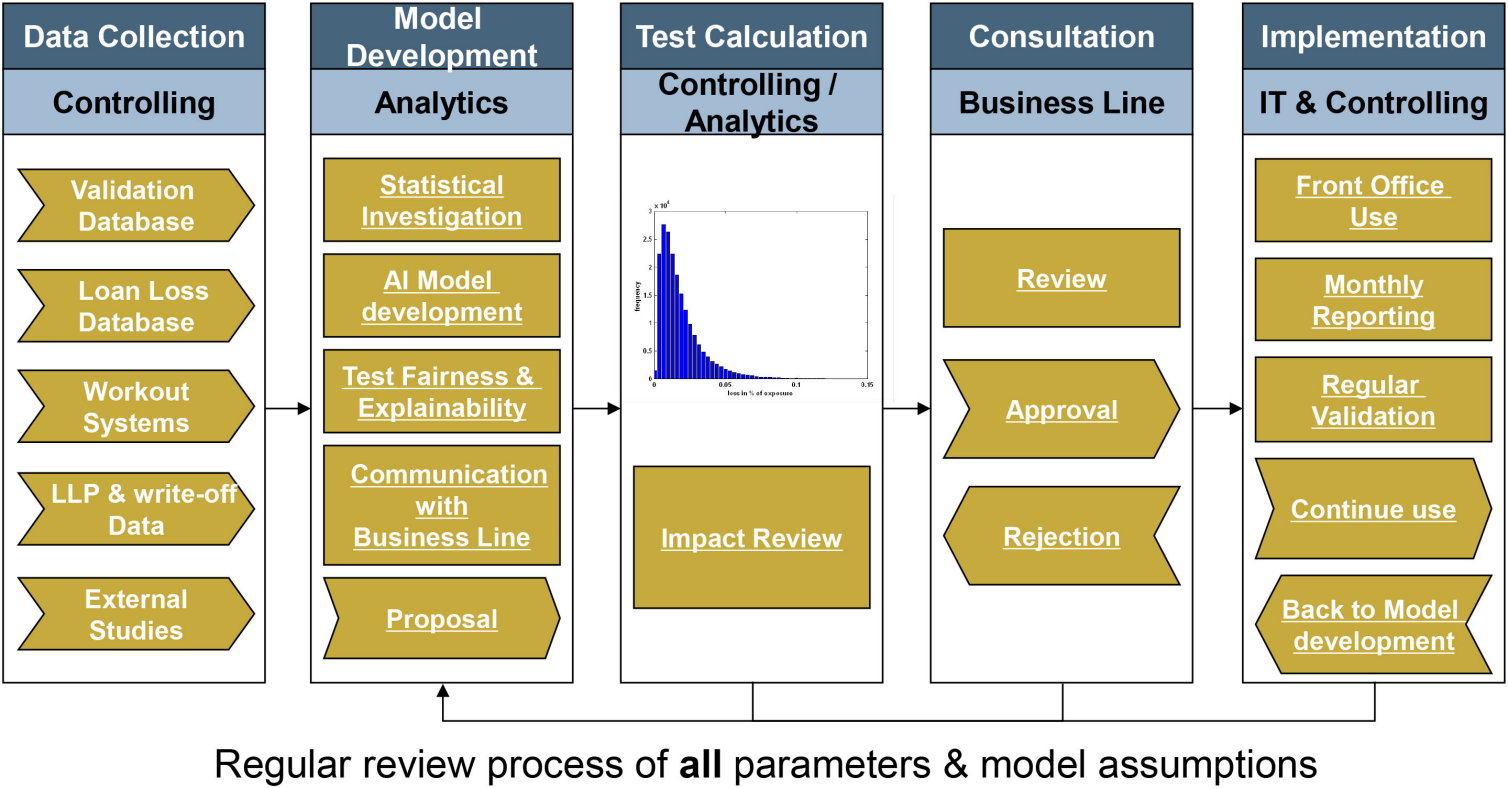
Simplified flowchart for a model development, application and validation cycle.

Figure 1 sketches a simplified flowchart for a model development, application and validation cycle. We have used Credit Scoring as example, but the process steps can also be applied to other types of AI model development.

Indulging in a reverie far beyond the financial services space, the authors believe that the promotion of fairness in AI could even help to increase societal fairness overall. Equal opportunities in terms of access to education, goods, services, and technology could be driven to a next and better level. In line with generally accepted ethical values, the use of AI systems should never lead to people being deceived or unjustifiably impaired in their freedom of choice. Additionally, fairness implies that AI practitioners should respect the principle of proportionality between means and ends and consider carefully how to balance competing interests and objectives.

## Addressing Trustworthy AI With Technology

Successful and ethical AI adoption means harnessing the amazing potential of AI while simultaneously implementing the principles of fair, trustworthy, and explainable AI. Some of these principles can be addressed by scaling digital technologies like computing platforms and then deploying smart models or specific algorithms. An entire industry including the corresponding academic and non-academic research is currently being built around this topic (see for example Liu et al., 2021 and references therein). The following paragraphs provide a list of examples of applications and technologies that should be considered to implement Trustworthy AI.

### Privacy-Preserving Technologies and Methods

Some technology is used to ensure that AI systems comply with privacy laws and regulations and meet societal norms for personal data and information privacy.

One technology area is differential privacy and multi-party computation for distributed/federated privacy-preserving machine learning. The term differential privacy is used to describe both the property of an AI system that looking at the output, one cannot tell whether any individual's data was included in the original dataset or not, and methods to establish this property. Federated learning enables multiple entities who do not trust each other (fully), to collaborate in training a Machine Learning (ML) model on their combined dataset; without actually sharing data.

Another methodological area is the AI-based generation of synthetic data with a wide range of applications, ranging from shortening cycles for quantitative risk modeling, minimizing the effort to comply with regulation, to accelerating development and testing processes. Smaller data sets can be amplified to improve model explainability. Adverse data scenarios can be tested to stress a model.

Synthetic data can also support the collaboration between FinTech companies and incumbent banks: for a closer collaboration, banks need to test the capabilities of third-party analytical providers. During this phase, they will not typically want to share their original data, but synthetic ones will provide a way forward.

Generative Adversarial Networks (GANs) are an approach to generative modeling using deep learning methods. Given a training data set, this technique learns to generate new data with the same statistical properties as the original training set. Since many approaches to generating synthetic data require computational power, the industry uses accelerated computing platforms for data generation.

Synthetic data emerges with the promise to provide an unlimited amount of representative, realistic training samples, that can be shared further without disclosing the privacy of individual subjects. However, in as much as e.g., GANs propagate the statistical properties of their training data, synthetic data must be assumed to be as flawed from a fairness perspective as actual data were and it should be considered unwise to use these data without further care, in the general case. Frameworks are needed to incorporate fairness constraints into the self-supervised learning process, that allows to then simulate an unlimited amount of representative as well as fair synthetic data. These frameworks will then provide the methods to govern and control privacy as well as bias within AI at its very source: the training data (see Tiwald et al., 2021).

### Technologies for Explainability and Transparency

There are many technologies that support the development of transparent and explainable AI (XAI). XAI can help to explain the ways in which less transparent AI models (“black boxes”) function to a certain extent. One approach to achieve model explainability of course is to use an interpretable model by design. However, easy to interpret models are often (multi-)linear models. The problem with this approach is that the level of simplification with which a reality is represented in the model is often very high, which means that model accuracy is frequently less than optimal. This is simply because complex higher-order relationships in the underlying data are not seen by the model.

According to AIRS (Artificial Intelligence/Machine Learning Risk and Security Working Group) (2020), examples of newer and perhaps relatively more accurate and sophisticated types of interpretable AI/ML systems include scalable Bayesian rule lists, Explainable Boosting Machines (EBMs), monotonic Gradient Boosting Machines (GBMs), various Bayesian or constrained variants of traditional AI/ML systems or other novel interpretable-by-design systems.

There is also a variety of model agnostic *post-hoc* approaches to model explainability. Well-known representatives are LIME (Local Interpretable Model-agnostic Explanations) and SHAP (SHapley Additive exPlanations). The latter is a game theoretic approach to explain the output of any machine learning model. It revalues an entire dataset (“global”) or individual instances (“local”) after removing features (“Explaining by Removing”). A sound introduction to responsible machine learning workflow with a focus on interpretable models, *post-hoc* explanation, and discrimination testing can be found in Gill et al. (2020).

The publication of Deutsche Bundesbank (2020) helps to assess the benefit and downsides of XAI: “There is a fundamental conflict between the implementation of ML, with its potentially highly non-linear behavior, and the demand for comprehensible linear explanations. Explanations put forward by XAI seem to be appealing and convenient, but they only show a limited picture of model behavior, from which it is hard to draw general conclusions. Thus, ML combined with an XAI approach cannot make the black box fully transparent, merely less opaque. Nonetheless, it seems to be helpful to use XAI to provide more reliable risk metrics for control processes. We propose a balanced approach with XAI methods to be tailored to the use case and to the stakeholders' demands. Further limitations of XAI methods should not be overlooked. Some methods require high computational power or only deliver minor insights into algorithms' behavior. XAI methods should support established and used risk control processes and be able to demonstrate effectiveness. If not applying XAI methods, control processes should be in place to compensate for limited transparency.”

The game-theoretic foundations, the supervisory attention on this particular approach (e.g., through publications by Bank of England) plus some other favorable features surely establish SHAP as one the standard approaches to understand and communicate feature importance at a global and local level. But it still is a compensation for missing transparency with ambitious control processes. The implementation of a SHAP approach itself establishes another model added to the workflow, with additional risks and costs. For example, it is known that strongly correlated features and other effects cause instabilities in SHAP evaluations, and SHAP can theoretically, at an additional effort, be “gamed” with the help of so-called scaffolding techniques, in the sense that the technique can be led to produce erroneous results. Also, as any other statistical model based on whichever type of co-incidental statistics, it does not explain causal relationships.

The downside of requiring time-consuming computations for SHAP revaluations, especially for large data sets, can be compensated by highly parallel processing, e.g., by High-Performance Computing (HPC) environments using GPUs. According to the IFC-BIS publication “Computing platforms for big data analytics and artificial intelligence” (see Bruno et al., 2020) “Central banks' experience shows that HPC platforms are primarily developed to ensure that computing resources are used in the most efficient way, so that analytical processes can be completed as rapidly as possible. […] A processor core (or “core”) is a single processing unit. Today's computers—or CPUs (central processing units)—have multiple processing units, with each of these cores able to focus on a different task. Depending on the analytical or statistical problem at hand, clusters of GPUs (graphics processing units, which have a highly parallel structure and were initially designed for efficient image processing) might also be embedded in computers, for instance to support mass calculations.” In real life, the superb computing power of GPU clusters can be read off the fact that the market for high performance GPUs is influenced both by crypto miners and the high-performance gaming community even in the retail sector. Industry corporates have long since been exploring GPU computing to solve high computing power demand situations.

An example for using interpretable machine learning techniques is a use case developed by Munich Re Markets, published in Jaeger et al. (2021). It is a robust, fast, and interpretable machine learning approach to diversified portfolio construction. The approach is useful for overcoming a profoundly serious problem in investment management: back-test overfitting and replication crisis. This is due to the few decades of historical data available in capital markets. The approach helps in developing robust correlation-dependent investment strategies as well as financial products. HPC platforms can accelerate the training and *post-hoc* interpretation tasks associated with this approach from days to minutes.

The model training quality and its interpretability can be amplified by artificially creating future market data scenarios that have never been observed before but nevertheless carry the statistical footprints of financial market behavior. An example of such a procedure is given in Papenbrock et al. (2021) who use an alternative approach to GAN to produce correlated market data. The approach is based on an evolutionary search heuristic which can be parameterized in a way that allows the adoption of normal as well as extreme scenarios, both unobserved in the past.

The evolutionary approach is surprisingly analogous and thus perfectly suitable for HPC platforms as well. With the help of Matrix Evolutions, it is possible to synthetically generate correlation scenarios that seem real(istic) but have not yet been observed during the short actual history of financial markets.

Like in a wind tunnel for airplanes, millions of scenarios can be tested, reflecting all kinds of wind turbulences that could occur before the plane gets certified for take-off and air travel. The same can now be applied to test and explain investment strategies. The approach is called “Matrix Evolutions” and basically creates synthetic correlation matrices in a controlled way to amplify the XAI model to be able to construct more robust investment portfolios or perform advanced market risk management operations^3^.

### Optimizing Trustworthy AI With Multiple Objectives

The previously presented application of Evolutionary Algorithms can be extended to optimize trustworthy AI. The idea is to formulate a number of objectives for AI/ML models that are supposed to be measurable characteristics of trustworthy AI models.

For example, we measure the explainability and fairness of a model with certain metrics and define those as two objectives of an optimization program. We also measure model accuracy as well as prediction speed (efficiency) as two additional objectives^4^. In the end, we have four objectives, which as a portfolio of objectives would typically contradict each other: for example, explainability and accuracy are often in conflict. This optimization program is quite complex as we are looking for a solution that finds the best compromise between several conflicting objectives. Mathematically, we need to search the efficient Pareto frontier in a multi-objective optimization problem. The real-world solution space is non-smooth and non-convex, whence we need a smart search strategy to heuristically solve this challenging problem. Only a heuristic optimization like an Evolutionary Algorithm can support this—a task which luckily lends itself very well to parallel processing. Only an HPC environment can support the solution finding process of such a setting in a reasonable amount of time. It will create a large number of models and will systematically adapt model parameters to develop a high-quality efficient Pareto frontier.

In the next step, each point in that frontier is displayed as a single line in a parallel-coordinate plot^5^. The coordinates correspond to the multiple objectives. The user can now decide which line to pick, which results in a model choice with certain levels in the objective functions. The user has the knowledge that this model choice exhibits specific levels of accuracy, explainability, efficiency, and fairness and that it is Pareto-optimal, meaning that it is the best compromise solution.

### Open-Source Data Science

Data science offers a powerful toolbox to approach the implementation of trustworthy AI, building its strategic technological foundation. Data engineering is a key step and involves data gathering, data preparation and preprocessing, data analytics, feature engineering, machine learning and data visualization.

Also, an architectural infrastructure is needed to build, manage, and monitor models through their entire lifecycle. When an algorithm is ready to be launched, MLOps is practiced between Data Scientists, DevOps, and Machine Learning engineers to transition the algorithm to production systems.

Financial Data Science and MLOps can be very time consuming, with the risk of ineffective utilization of expensive expert resources. Therefore, it is particularly important to build an infrastructure and software stack that leverages those resources to the best possible efficiency.

Python is the most-used language in Data Science today. Libraries like NumPy, Scikit-Learn, and Pandas have changed how we think about accessibility in Data Science and Machine Learning.

However, while great for experimentation, PyData tools still lack the power that is necessary for enterprise-scale workloads. This leads to substantial refactoring to handle the size of modern problems, increasing cycle time, overhead, and time to insight.

Quintessentially, to keep up with the forefront of development, an end-to-end accelerated infrastructure is needed that saves time and fully supports the capabilities of the Data Science and model management teams. Suites of open source software libraries exist that exploit parallelism in computation, node cluster scaling and high-bandwidth memory speed to power through big data processes and combine them with user-friendly Python interfaces^6^.

Unsupervised learning like clustering, graph network analysis and visualization are especially useful tools as they require no expensive data labeling. They reveal the hidden relationships and links within the data sets and can visualize and interactively analyze the data, using the human superpower of visual analytics.

The increasing size of graphs to be visualized calls for tools outside the classical desktop visualization paradigm. To this end, parallel and distributed computing can help a pre-computation phase for open source software libraries multiscale visualization or graph drawing.

### Further Reading and Resources for This Chapter

The topics model risk management, trustworthy AI, AI governance, AI certification and AI incident management are currently being discussed in the industry and at regulators/supervisors. Some institutions are developing digital platforms that address these issues technologically^7^.

The area of “algorithmic auditing” is quickly emerging and becoming an important aspect in the adoption of machine learning and AI products in enterprises. Companies are now incorporating formal ethics reviews, model validation exercises as well as internal and external algorithmic auditing to ensure that the adoption of AI is transparent and has gone through thorough vetting and formal validation processes. However, the area is new, and organizations are realizing that there is an implementation gap on how algorithmic auditing best practices can be adopted within an organization.

One important notion for the adoption of AI algorithms into operational decision processes is the concept of assurance. According to Batarseh et al. (2021), this is “a process that is applied at all stages of the AI engineering lifecycle ensuring that any intelligent system is producing outcomes that are valid, verified, data-driven, trustworthy and explainable to a layman, ethical in the context of its deployment, unbiased in its learning, and fair to its users.” The paper further delivers an overview of the AI assurance landscape, a method review and some recommendations and outlook on the field, highlighting the need for multi-disciplinary collaborations.

The Frankfurt Institute for Risk Management and Regulation is also engaged in several GAIA-X^8^ projects in the Finance & Insurance Data Space. They are related to addressing Trustworthy AI with technology, among other objectives. The project FAIC (Financial AI Cluster) is focused on the implementation of technologies to support trustworthy, explainable AI, as well as algorithmic auditing and sandboxes/experimentation facilities^9^. FAIC activates an ecosystem and an entire tech industry around trust-creating technologies for AI model building, controlling, and auditing with innovative algorithms and HPC/AI computational infrastructure, e.g., for synthetic data generation, federated learning, and confidential computing. An industry with new services and applications for AI risk management and MLOps, supporting the implementation of trustworthy AI, will emerge, and platforms will be built for “Computational Trustworthy AI' and “Computational AI Assurance”^10^. The project's foundations had been laid out in the European Union's Horizon 2020 research and innovation program called FIN-TECH^11^.

## A European Use Case For Explainable AI in Credit Risk Management

The purpose of this chapter is to walk the reader through the steps of a practical *post-hoc* SHAP-value-based XAI example that enhances the Credit Risk Management process. We demonstrate its capabilities, value adds, and effectiveness.

The example is based on the best rated use case of the EU Horizon2020 project FIN-TECH on Explainable AI. FIN-TECH is a FINancial supervision and TECHnology compliance training program, which draws on contributions of

- Fintechs and fintech hubs who have a detailed understanding of business models based on financial technologies;
- Regulators and supervisors who have a detailed understanding of the regulations and risks that concern financial technologies;
- Universities and research centers which have a detailed understanding of the risk management models that can be applied to financial technologies.

One key focus area was to analyze what inhibits AI from scaling across Europe's financial services industry. Several supervisors and central banks had expressed their concern about black box AI models and the related impending inability to control them. The FIN-TECH project identified that this could be a potential inhibitor of industry growth, investment, and innovation in AI. It therefore took the opportunity to search for ways to overcome the explainability gap of AI models, and to produce standard use cases on explainable AI.

One FIN-TECH use case addresses a credit risk management example and uses SHAP values to identify the most important variables for decision making in a trained AI/ML model. This use case is inspired by a model published by Bracke et al. (2019) from the Bank of England. Above that, the approach further explores the similarity of explanatory data of the credit portfolio constituents using unsupervised learning approaches like clustering in graph analytics. The purpose is to not only be able to analyze the explanatory data either locally or globally, but also in groups or clusters, where every cluster consists of portfolio constituents with very similar explanatory data. In this way it is possible to get an in-depth understanding of the functional details of a trained model, to potentially debug it, and to control it, as we will demonstrate in this section.

**Figure 2:**
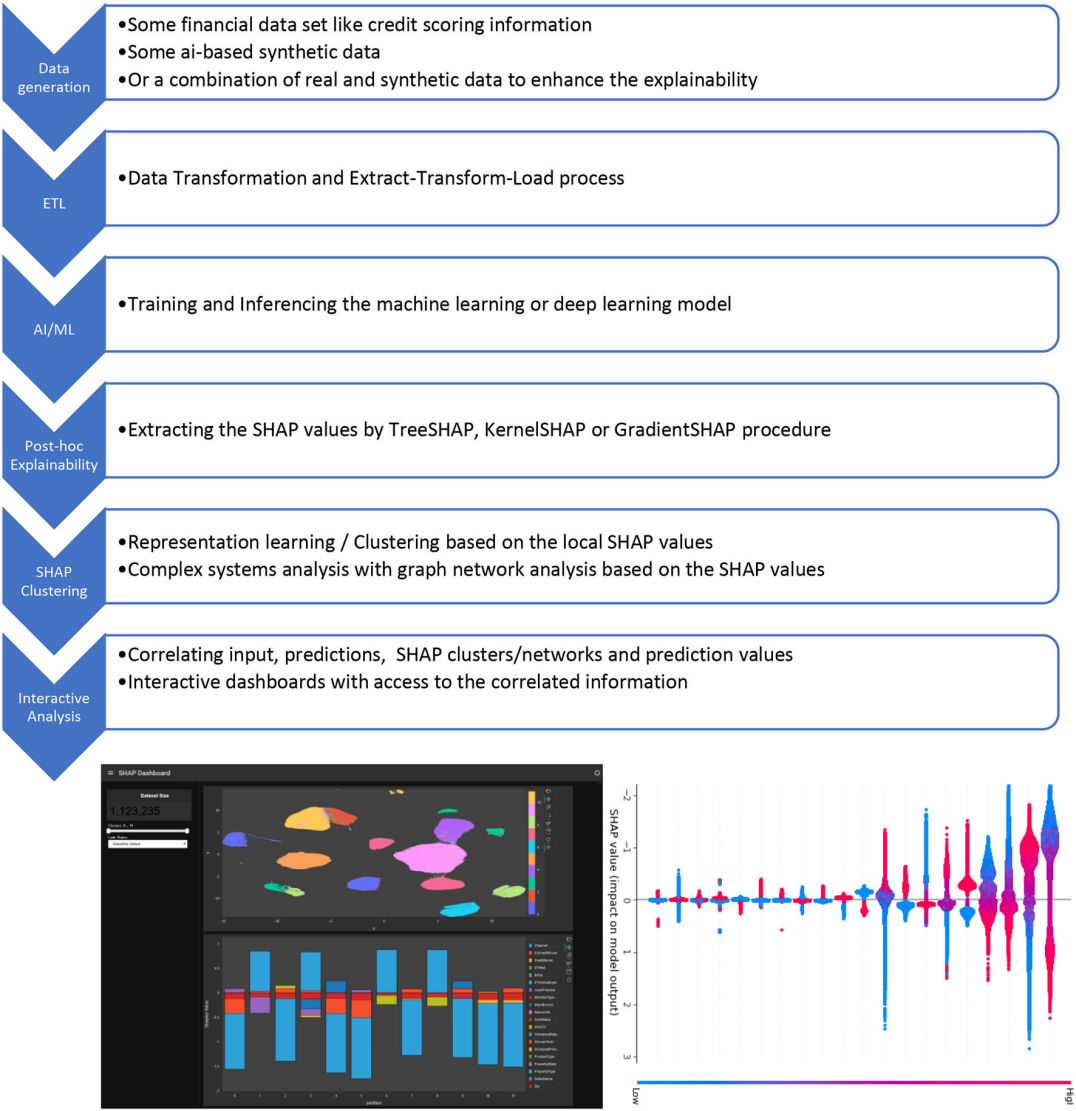
Description of the end-to-end workflow for an application to interactively analyse post-hoc explainable AI information. The dashboards have been produced with Plotly.

We call the approach “SHAP clustering” (the concept is explained below and in the references), it was published as Bussmann et al. (2020) and was included in the FIN-TECH workshop series with supervisory authorities, central banks and the financial service companies and fintechs all across Europe. The project evaluation system identified that this AI/ML use case was among the most successful and popular ones. It is very straightforward to use and the workflow and combination of algorithms could be applied to a number of AI/ML applications. The target area of application of the proposed workflow and approach are risk management, assessment and scoring of credit portfolios in traditional banks, as well as in “fintech” platforms for p2p lending/crowdfunding, but also in constructing diversified investment portfolios. Figure 2 has an overview of the steps:

Description of the end-to-end workflow for an application to interactively analyse *post-hoc* explainable AI information.

The specialty and added value of this approach is that the *post-hoc* explainability information is arranged into clusters of similarity (or user-defined groups), and that these data can be aggregated on each cluster/group level. Further, the data can be correlated with the input features and predictions. Lastly, all this information can be made available in huge interactive dashboards to slice, dice, and correlate all data as the user requires and finds useful. Below, we will discuss some use case scenarios.

Another added value is that each of the steps is GPU-accelerated, and the entire computation can be performed in GPU memory, avoiding costly copying steps between CPU and GPU. This makes the approach very efficient for large, enterprise-scale data sets and workloads. Explainable AI needs to be quickly available for consumer fintech applications. Explanations about different factors may be needed within milliseconds, depending on the use cases^12^. GPU-acceleration enables customer service representatives to explain automated financial decisions—like loan acceptances—and build trust with transparency about the process. In summary, the case presented is relevant for companies and enterprises that want to implement explainable AI at the enterprise level—for a XAI-effective Credit Risk Management process, for example.

The original data set used in the FIN-TECH project was rather small compared to the real-life data sets of some financial institutions. It was reasonable to test the model on a larger data set to understand the implications in production environments at a realistic scale. A team implemented the entire workflow in RAPIDS^13^ to be able to process large amounts of data within a reasonable time. RAPIDS is a suite of open-source Python libraries that can speed up data science workflows using GPU acceleration. The team picked a Fannie Mae data set similar to a related blog on explaining and accelerating machine learning for loan delinquencies^14^.

The presented machine-learning-based and visual procedure proposed in the approach is generally capable of processing the outcomes of any given (arbitrary) AI/ML model in a *post-hoc* explainability approach. Thus, it provides more insight, control, and transparency to any trained, potentially black box machine learning model. It utilizes a model-agnostic method aiming at identifying the decision-making criteria of an AI system in the form of variable importance (individual input variable contributions) with applications in credit risk assessment and management as well as in other (financial) areas. A key concept is the Shapley value decomposition of a model, a pay-off concept from cooperative game theory. So far, the Shapley value decomposition is the only XAI (explainable AI) approach rooted in an economic foundation. The approach offers a breakdown of the contributions of individual variables to the forecast outcome, typically some predicted probability. This means that after application of the method, every data point (e.g., a credit or loan customer in a portfolio) is not only represented by input features (the input of the machine learning model) but also by variable contributions to the prediction of the trained machine learning model. Practitioners usually analyse a local data point looking at the Shapley values or they aggregate Shapley values by averaging globally to learn something about the total model in terms of its feature importance.

The explanation values are also used for visual mapping based on dimensionality reduction techniques [such as Principal Component Analysis, Multidimensional Scaling, t-SNE (t-Distributed Stochastic Neighbor Embedding)], or for representation learning like clustering and graph-analytics (such as community detection). This enables to analyse the data on an intermediate level between local and global. Those data-driven, learned representations reveal segmentations of data points (customers), where each of the segments (or clusters) represents a group with very similar decision making information (i.e., similar importance of similar features) whereas data points in other clusters exhibit comparatively different, but again homogeneous decision making structures. (Hierarchical) Clustering and especially graph theory and network analytics are very well-suited to study these kinds of complex systems based on the decision making data of an AI/ML model. These systems are characterized by emergent, self-organizing properties.

The approach treats the variable contribution outcome of a (black box) machine learning model as a complex system, and further analyses its properties by graph theory and cluster analysis. In this way, the user gets a better and deeper understanding of what exactly a black box machine learning algorithm has learned. The following phenomena inside the black box model can be analyzed and understood: trends, anomalies, hot spots, emergent effects, and tipping points. Since the methodology is model agnostic, it can be applied to any machine learning model. This also enables a comparison of several machine learning models trained on the same data. The complex system of decision-making mechanisms that belong to a series of machine learning models can be compared to each other.

The proposed SHAP clustering approach enables a better understanding of model explanation information. Here are some use case scenarios:

- Groups or clusters of data points express “similar” decision making of the underlying, to-be-explained ML/AI model. These clusters summarize the mechanics of the model and represent types of decision making of the model, thereby effectively introducing a topology (i.e., a notion for proximity and distance) on the space of decision-making structures used by the non-linear model in view. Users get a better understanding of what the model has learned, to verify it, and to check plausibility.
- Data points at the intersect between clusters point to fuzzy decision making, which can be further investigated.
- A cluster with almost equal amounts of predictions for default and non-default could point to bugs or problems in the machine learning model. It could be checked whether or not the corresponding data points are actually indifferent, or whether the model exhibits buggy decision making on the corresponding data subset or “region” (in the sense of the above loosely mentioned intuitive notion of a topology) of the model.
- Customer segmentation: taking this technique as a means to segment portfolio data would render a novel approach to portfolio segmentation. Namely, the data points (credit customers and loans) could not only be clustered by their input variables (representing clusters of similarity of the customers) but also by their variable contributions in the decision making (representing the way in which they lend themselves to distinct types of decision making). This new clustering incorporates the “intelligence” of the machine learning model (the mapping of input variables to the default labels). Customers in the same cluster provoke similar decision making by the underlying, to-be-explained, machine learning model, e.g., about how and whether they default or not. In that sense, one could use a black-box model to learn a prediction on the portfolio, then use our XAI technique to find sub-portfolios where there is large similarity in decision making by the original machine learning model, and then train simpler (possibly even linear, or transparent-by-design) models on each such segment to maximize explainability.

The decisions of any reliable model must be informed decisions, and there must be a human-in-the-loop oversight that assigns accountability to a clearly defined function in the organization of the corporate that deploys the model. The SHAP clustering approach enables its user to understand why a particular decision was made. The “why” is not causal, but is expressed as a set of numeric contributions of input variables (which variables are responsible for the result). The user can look at a specific data point in the portfolio and see the corresponding input variables, the contributions of these variables to the prediction as well as the prediction itself. As one effect, a more human-based, plausible explanation can arise to reconcile the machine-based decision with a human narrative “this makes sense.” The model can be better controlled, as it delivers feedback on how it reaches each singular decision, and thus all decisions both on global level (global variable importance) and on a local level (data points). The clustering step even delivers the variable contributions for the members of that specific cluster, i.e., there is an intermediate level between the global and the local, which corresponds to a partition of the input portfolio of customers. The user could identify properties of this group of customers based on the input variables to understand how the decision-making works for this group of customers. All these analytics capabilities and tools plus the interactive visual exploration enable the user to better understand the outcome of an otherwise entirely black box model. Better understanding leads to more effective control.

To ensure traceability, a mechanism of documentation to the best possible standard should be incorporated. Be it, for example, the documentation of data sets, data labeling, or the decisions made by the AI system. The SHAP approach allows its user to trace back and document the variable contributions to decision making. The clustering of SHAP information is one of the new pieces of information added by the approach, so this can be used to enrich the traceability and documentation. Also, the steps to improve the model based on the new information could be documented.

According to the Guidelines of the HLEG, explainability concerns the ability to explain both the technical processes of an AI system and the related human decisions. The explanation must be understandable to all users (to a certain extend).

If this is the task, then what should the term explainability mean? Every human being will favor their own interpretation when a model is “fully explained” in their respective view. Irrespective of this fact, we enumerate a few partly provocative and incomplete definitions of the term. A model could be called “explained” when a majority of the audience of the attempt to explain (potentially represented by a committee) would agree that:

- Further information would not decrease their urge to ask “why” any more
- They feel sufficiently enabled to explain the AI-made decisions to independent third parties
- The documentation/visualization makes its functioning clear or easy to understand.
- XAI “proves the work,” i.e., the explanation enhances their trust in the respective AI so as to accelerate adoption.
- The result ensures compliance with expanding regulatory and public expectations and
- Fosters trust.

It is true that the SHAP approach delivers an explanation of the global and local decision making of a black box machine learning model, so it explains the AI model on all levels. However, our extension facilitates further analysis of the explanations, for example in context with other local decision mechanisms and data points. Thus, a richer set of information about the decision-making mechanism is given. This could lead to a situation where a human narrative arises and a “story that is plausible” can be delivered. In this way, the machine decision making is connected to human decision making. However, as mentioned before, it is not an explanation in terms of a “causal why.”

Even as it becomes clear from the audience-dependent concept we propose for the word “explainability,” an important aspect of explainability is the clarification of the audience. The audience of a model explanation is manifold:

- Model builders
- Model checkers
- Compliance and governance officers
- Risk managers
- Product owners
- Senior managers
- Supervisors
- Clients/customers

The SHAP (cluster) information can be understood by the data scientists or model developers and model validators. Most other relevant people in a bank or fintech company would (we claim) understand it with sufficient training. The same should apply to internal and external auditors and supervisors. For customers/and clients it may be sufficient to mention which variables are most important (the client should probably be informed about the reason for a decision/rejection) or what a client could do to improve certain variables to get a positive decision. The SHAP information delivers a consistent and accurate view and language to describe an AI model. However, it cannot be mentioned often enough that it is also only just a model and that correlation is not causation.

The presented approach can also be used to enhance the Z-Inspection®^15^ process to assess trustworthy AI, especially in the conceptual cluster of transparency/explainability/intelligibility/interpretability^16^. The Z-Inspection® process has the potential to play a key role in the context of the new EU Artificial Intelligence (AI) regulation.

Our approach draws attention to the problem of a black box AI, which does not in itself provide an immediate possibility to identify the input and decision-making criteria of such systems. In such situations, it is often difficult to know

(i) how reliable the inferred relationship between input and output is and
(ii) which causality exists between them. This is called the explanatory gap of AI.

It is understandable to the authors why banking supervisors would oppose the use of such unexplained black box AI models in the financial sector, at least in areas of application where they themselves are responsible for monitoring and challenging the regulatory compliance of such models. They must assume that an unexplained AI model is not only unexplained to them, but that also the developers of the model and its users lack understanding of the model's driving forces and rationales. However, this should not lead to a general rejection of the use of AI models. In our view, supervisors will have to further adjust their approaches and potentially further transform their skills to also escort the introduction of AI/ML in banking, including the demand for appropriate XAI setups where necessary. On the other hand, banks and other financial institutions will need to expect that supervisors will demand sound explanations of what their AI/ML systems actually do, and what their business purpose is. Approaches like the SHAP clustering are a contribution to both: banks and supervisors.

## Conclusion

Based on the discussions at the Round Table AI at FIRM (Frankfurt Institute for Risk Management and Regulation), the activities in the Gaia-x project FAIC as well as the work around building the “European Use Case for Explainable AI in Credit Risk Management” we have identified seven conclusions and takeaways that express our persepctive on “Financial Risk Management and Explainable, Trustworthy, Responsible AI”:

1. There need to be general principles, requirements, and tests to control model risk and fitness-for-purpose for each model. Particularly because AI is not a fixed category, we are talking about a spectrum of mathematical models of varying complexity, of which gradually increasingly complex ones are becoming feasible. The mentioned governance elements (principles, requirements, tests to control model risk etc.) should focus on models' respective purposes, influence on human lives, and business impact, rather than model design or complexity. To satisfy these requirements of course, special tests will be necessary for more complex or even dynamic models. This holds true especially for the implementation of those models and their utilization in a scaling enterprise production environment.
2. To this end, it will be necessary and useful to combine the expertise and approaches of classical risk management and governance with those of data science and AI knowledge.
3. Many aspects of AI governance, algorithmic auditing, and risk management of AI systems can be addressed with technology and computing platforms.
4. In fact, an entire industry is about to emerge in this area. Many of the necessary techniques essentially consist of the use of somewhat less complex and more transparent models in their own right, with associated cost for maintenance and operation, and with inherent (more indirect and smaller) risks to operate them. Hence, there will always be residual risk and consequentially a need for human oversight. The level of residual risk should be covered via OpRisk Management (IT risk, mal-decisioning risk, reputational risk) and by AI incident management or AI model insurance.
5. Explainability, interpretability, and transparency of models, data, and decision making will be key to even enable an appropriate possibility to manage remaining model risks (“Explaining Explainable AI”). All three need to be directed toward the internal stakeholders of financial institutions, but—depending on model purpose—also toward the outside world, particularly to clients/consumers and supervisors. Each stakeholder needs to be informed about the model aspects in a different and specific way. There are technologies and experts to support interfacing the different domains involved.
6. One aspect of the “Explainable AI” agenda is to enable the fairness of AI decision making or decision support from a societal perspective (linked to the ESG agenda). The associated fairness considerations, starting from the need to explicitly define a notion of fairness and enable its implementation and ongoing validation, are by no means exclusive to AI modeling techniques. They pertain to classical decision making to the same extent; however, due to their lack of innate transparency, the cost of fairness will be higher for AI models. This should be taken into account in the business decisions around the choice of model design.
7. We propose that the final decision about which model should be used, which one needs to be reviewed, and which models should be discontinued, should always be made by a human being. This ensures that the responsibility resides with the respective human decision maker, but is also an important control for drift in self-learning models.

## Data Availability Statement

The original contributions presented in the study are included in the article/supplementary materials, further inquiries can be directed to the corresponding author/s.

## Author Contributions

All authors listed have made a substantial, direct, and intellectual contribution to the work and approved it for publication.

## Funding

This work relies on the support from the European Union's Horizon 2020 research and innovation program FIN-TECH: A Financial supervision and Technology compliance training programme under the grant agreement No. 825215 (Topic: ICT-35-2018, Type of action: CSA).

## Conflict of Interest

SF-M was employed by Bain & Company. BH was employed by Ernst & Young. JP was employed by NVIDIA GmbH.

## Publisher's Note

All claims expressed in this article are solely those of the authors and do not necessarily represent those of their affiliated organizations, or those of the publisher, the editors and the reviewers. Any product that may be evaluated in this article, or claim that may be made by its manufacturer, is not guaranteed or endorsed by the publisher.

## Footnotes

^2^Ethics embeds the following dimensions: Privacy, Avoidance of Bias, Fairness, Explainability, Transparency, Impact.

^3^This example shows that “The Future of Explainable AI rests upon Synthetic Data” (https://mostly.ai/2021/03/25/the-future-of-explainable-ai-rests-upon-synthetic-data/).

^4^This is similar to the example in https://medium.com/turintech/optimising-ai-with-multiple-objectives-why-ai-efficiency-is-critical-for-scaling-ai-turintech-6638f3d77dc6.

^5^See for example https://en.wikipedia.org/wiki/Parallel_coordinates or https://plotly.com/python/parallel-coordinates-plot/.

^6^Example are Spark, Dask, and RAPIDS.ai.

^7^See for example https://www.quantuniversity.com/publications.html and https://www.bnh.ai/public-resources.

^8^Gaia-X represents the next generation of data infrastructure: an open, transparent, and secure digital ecosystem, where data and services can be made available, collated, and shared in an environment of trust (https://www.gaia-x.eu/).

^9^The blog article “The innovation potential of GAIA-X: Technologies for the trusted use of artificial intelligence in the finance sector” has more details.

^10^This development could be confirmed in the Global Webinar Series: “AI, Explainability, and Trustworthiness in Financial Services” presented by World Economic Forum (WEF), World Alliance of International Financial Centers (WAIFC), and NVIDIA.https://waifc.finance/news/new-global-webinar-series-on-artificial-intelligence-in-finance/.

^11^The project has received funding from the European Union's Horizon 2020 research and innovation program under grant agreement no. 825215 (topic ICT-35-2018, Type of action: CSA).This content reflects only the author's view and the Commission is not responsible for any use that may be made of the information it contains (https://fintech-ho2020.eu/).

^12^See this blog by Fiddler Labsi: https://blogs.nvidia.com/blog/2020/08/04/fiddler-labs-ai-model-explainability.

^13^http://www.rapids.ai/.

^14^The blogs can be viewed at: https://developer.nvidia.com/blog/author/jochenpapenbrock/. The GPU-accelerated Python code is available at: https://github.com/NVIDIA/fsi-samples/tree/main/credit_default_risk.

^15^http://z-inspection.org/.

^16^See Zicari et al. (2021).
